# Building an improved transcription factor-centered yeast one hybrid system to identify DNA motifs bound by protein comprehensively

**DOI:** 10.1186/s12870-023-04241-8

**Published:** 2023-05-04

**Authors:** Wang Jingwen, Wang Jingxin, Zhu Ye, Zhu Yan, Liu Caozhi, Chen Yanyu, Zeng Fanli, Chen Su, Wang Yucheng

**Affiliations:** 1 Key Laboratory Forest Tree Genetics & Breeding of Liaoning Province, College of Forestry, Shenyang Agricultural University, Shenyang, 110866 China; 2 State Key Laboratory of Tree Genetics and Breeding, Northeast Forestry University, Harbin, 150040 China; 3 Nanjing Ruiyuan Biotechnology Company Limited, Nanking, 210000 China

**Keywords:** Chromatin immunoprecipitation, DNA-protein interaction, EMSA, ERF, High-throughput sequencing, MEME analysis, Transcription factor-centered yeast one hybrid

## Abstract

**Background:**

Identification of the motifs bound by a transcription factor (TF) is important to reveal the function of TF. Previously, we built a transcription factor centered yeast one hybrid (TF-Centered Y1H) that could identify the motifs bound by a target TF. However, that method was difficult to comprehensively identify all the motifs bound by a TF.

**Results:**

Here, we build an improved TF-Centered Y1H to comprehensively determine the motifs bound by a target TF. Recombination-mediated cloning in yeast was performed to construct a saturated prey library that contains 7 random base insertions. After TF-Centered Y1H screening, all the positive clones were pooled together to isolate pHIS2 vector. The insertion regions of pHIS2 were PCR amplified and the PCR product was subjected to high-throughput sequencing. The insertion sequences were then retrieved and analyzed using MEME program to identify the potential motifs bound by the TF. Using this technology, we studied the motifs bound by an ethylene-responsive factor (BpERF2) from birch. In total, 22 conserved motifs were identified, and most of them are novel cis-acting elements. Both the yeast one hybrid and electrophoretic mobility shift assay verified that the obtained motifs could be bound by BpERF2. In addition, chromatin immunoprecipitation (ChIP) study further suggested that the identified motifs can be bound by BpERF2 in cells of birch. These results together suggested that this technology is reliable and has biological significance.

**Conclusion:**

This method will have wide application in DNA-protein interaction studies.

**Supplementary Information:**

The online version contains supplementary material available at 10.1186/s12870-023-04241-8.

## Background

The interaction between a protein and DNA plays an essential role in many molecular and cellular processes, such as transcriptional regulation, transcription, DNA modifications, chromosome segregation at mitosis, DNA replication and repair, cell cycle progression, chromosomal stability, and epigenetic silencing [1]. This interaction also plays a key role in gene expression regulation. Transcription factors (TFs) bind to motifs in the promoters of genes to activate or silence them. Therefore, the identification and characterization of TFs and their interacting cis-acting regulatory elements are essential to understand gene expression regulation [2, 3].

To date, several methods to identify the interactions between DNA and TFs have been developed. These methods can be classified into two complementary approaches. One is the protein-to-DNA (TF–centered) approach, which uses a TF of interest to determine the DNA targets that it binds. The TF-centered approach includes the methods such as chromatin immunoprecipitation (ChIP), systematic evolution of ligands by exponential enrichment (SELEX), and the DNA adenine methyltransferase identification (DamID) [4, 5]. The other is the DNA-to-protein (Gene-centered) approach, which uses one or more regulatory DNA elements of interest and determines which TFs can bind to them. Among the gene-centered approach methods, Yeast one hybrid (Y1H) is an important technology to identify protein-DNA interactions. It has become an important technique to detect physical interactions between the regulatory DNA elements and TFs.

The classical Y1H system has two components. One component is a reporter construct harboring the DNA sequence of interest cloned upstream of a reporter gene that can be detected; the DNA of interest is called the “bait”. The other component is an effector construct that contains a fusion between a yeast transcription activation domain (AD) and a TF of interest, and the TF is usually called the “prey”. Both bait and prey components are introduced into a suitable yeast strain, and the AD will induce reporter protein expression when the TF can bind to the DNA of interest [6].

However, the classical Y1H method can only determine the TFs that bind to the DNA sequence of interest, and cannot screen the motifs bound by a TF of interest. Previously, we built a TF-centered yeast one hybrid system (TF-Centered Y1H) that also contains two components, the prey constructs harboring a random DNA insertion library, and the bait construct comprising a cloned TF of interest [7]. This method can determine the motifs bound by a target TF. However, it suffered from a disadvantage of low efficiency in determining the motifs, and thus the positive clones have to be sequenced one-by-one using Sanger sequencing technology. In addition, TF-Centered Y1H cannot identify the frequency of different bases at certain position of motif, as in ChIP-Seq. Therefore, it is difficult to screen the motifs of a TF comprehensively using this method.

In the present study, we first constructed a high-quality prey library, and then combined this method with high-throughput sequencing technology to screen the motifs bound by a TF of interest. We found that this method could identify the motifs that bind a TF efficiently, and could be used to determine the frequency of different bases at certain position of motif. Therefore, this method will have a wide application in the complete determination of the motifs that are bound by a TF.

## Results

### The principle and procedure of the improved TF-Centered Y1H

**Fig. 1:**
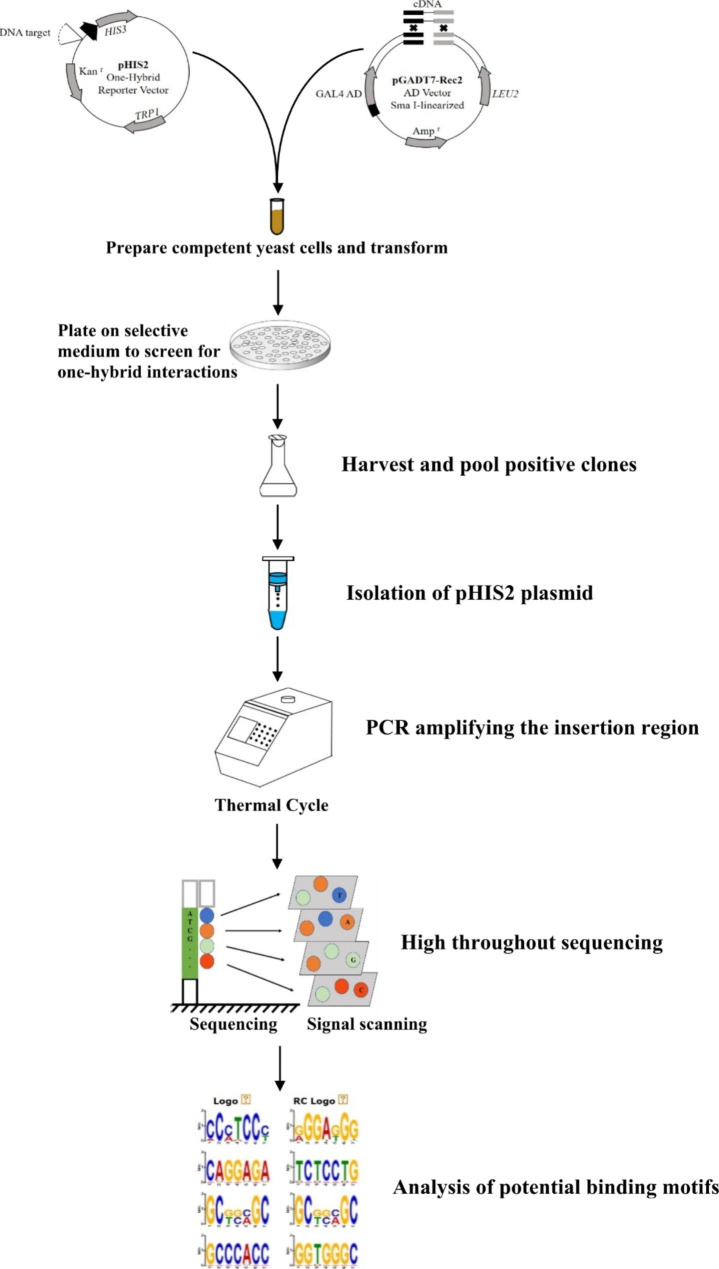
The principle and procedure of the improved Transcription Factor-Centered yeast one hybrid (TF-Centered Y1H). A 7 bp random DNA sequence was inserted into pHIS2 at the digestion site of Sma I to form a library using the yeast recombination method, which was served as prey. The studied TF was cloned into pGADT7-Rec2 and was used as bait. After Y1H screening, all the positive clones were harvested and pooled together for pHIS2 isolation. The truncated pHIS2 containing the insertion sequence (125 bp in length) was PCR amplified, and the PCR products were analyzed using high-throughput sequencing. The insertion sequences were retrieved from the reads. The reads were further analyzed using MEME or other programs to find the conserved DNA motifs, which are potentially bound by the aim TF

The classic Y1H using a DNA sequence cloned into pHIS2 as the bait, and a cDNA library cloned into pGADT7-Rec2 as the prey library. Conversely, in TF-Centered Y1H, a random DNA sequence library in pHIS2 was generated as the prey library, and the aim TF was cloned into pGADT7-Rec2 as the bait (Fig. 1). Y1H hybridization was performed, and the positive clones were harvested and pooled together. Then, the vector of pHIS2 was isolated. The insertion region of pHIS2 was PCR amplified using pHIS2 library as the template, and the PCR product was analyzed using high-throughput sequencing. The insertion sequences were retrieved from reads and further analyzed using MEME or other program to identify the DNA motifs potentially bound by the studied protein (Fig. 1).

### Construction of the random DNA insertion prey library

The random DNA insertion library was constructed as follows. The SsInS (single strand insertion sequence) sequence and the complementary primer (CP) for SsInS was designed to synthesize the double stranded DNA using Taq enzyme with one cycle only (Table 1). Then, the synthesized DNA was infused with linearized pHIS2 to construct the prey library using the yeast recombination method. After selection on synthetic defined (SD) yeast medium (without Histidine, Leucine and Tryptophan), we further randomly selected 30 plates, and each plate randomly selected 1 clone to determine whether the insertion had been correctly inserted in pHIS2 using Sanger sequencing. The results showed that all the selected colonies contained the random insertion sequence with correctness position, indicating that the SsInS had been successfully integrated into pHIS2. The colonies that grew on plates (12 cm in diameter) were washed using a yeast extract peptone dextrose (YPD) liquid medium. In total, 300 plates were washed containing about 150,000 colonies. The 7 nt random insertion library contained 4^7^ = 16,384 colonies; therefore, 150,000 colonies should include all the sequences of 7 nt random insertion with high probability. These results indicated that the random insertion library was constructed successfully.

**Table 1:** The primers used to construct the prey library and PCR primers used to amplify the insertion sequences

Names	Sequence (5′ − 3′)
SsInS	TGTAAAACGACGGCCAGTGAATTGTAATACGACTCACTATAGGGCGAATTCCCNNNNNNNNNGGGGAGCTCACGCGTTCGCGAATCGATCCGCGGTCTAGAAATTCCTGGCATTATCACATAATG (the insertion of “NNNNNNN” was underlined)
CP	CATTATGTGATAATGCCAGG
P1	TGTAAAACGACGGCCAGTG
P2	CATTATGTGATAATGCCAGG
BpERF2-F	GTATCAACGCAGAGTGGCCATTATGGCCGGGATGTGTGGGGGTGCTATCAT
BpERF2-R	ATTCTAGAGGCCGAGGCGGCCGACATGCTAATACATGAGCTTCAGTT

### Screening and identification of the motifs bound by BpERF2

For analysis of the motifs bound by BpERF2, pGADT7-Rec2-ERF2 was transformed into yeast harboring the random DNA insertion library (prey library), and selected SD/-Leu/-Trp/-His medium supplied with 30 mM 3-AT. The positive colonies were harvested by spreading on plates and pooled together for isolation of pHIS2, then the truncated DNA of pHIS2 containing the insertion sequence were PCR amplified. The PCR products were sequenced on an Illumina Hiseq 2000 instrument. In total, after removing low-quality sequences, 2,134,920 high-quality reads were obtained. Of them, 2,031,510 (95.16%) reads were successfully identified and used for further analysis. The raw data are available at https://figshare.com/articles/dataset/Short_DNA_sequence_recognized_by_ERF2/19164662, and the DOI number was 10.6084/m9.figshare. 19,164,662.v1. The insertion sequences were extracted from the clean reads, and were subjected for further analysis.

ERF family members can bind to a GCC-Box (“AGCCGCC”) and a DRE/CRT motif (“A/GGCCGA”); therefore, we first screened the GCC-Box and DRE/CRT sequences among the insertion sequences. The results showed that there were 89 reads representing the GCC-Box, and 294 reads representing the DRE/CRT motif (data DOI: 10.6084/m9.figshare. 19,164,662.v1). These results indicated that the GCC-Box and DRE/CRT motifs, which are known to bind BpERF2, had been successfully identified.

**Fig. 2:**
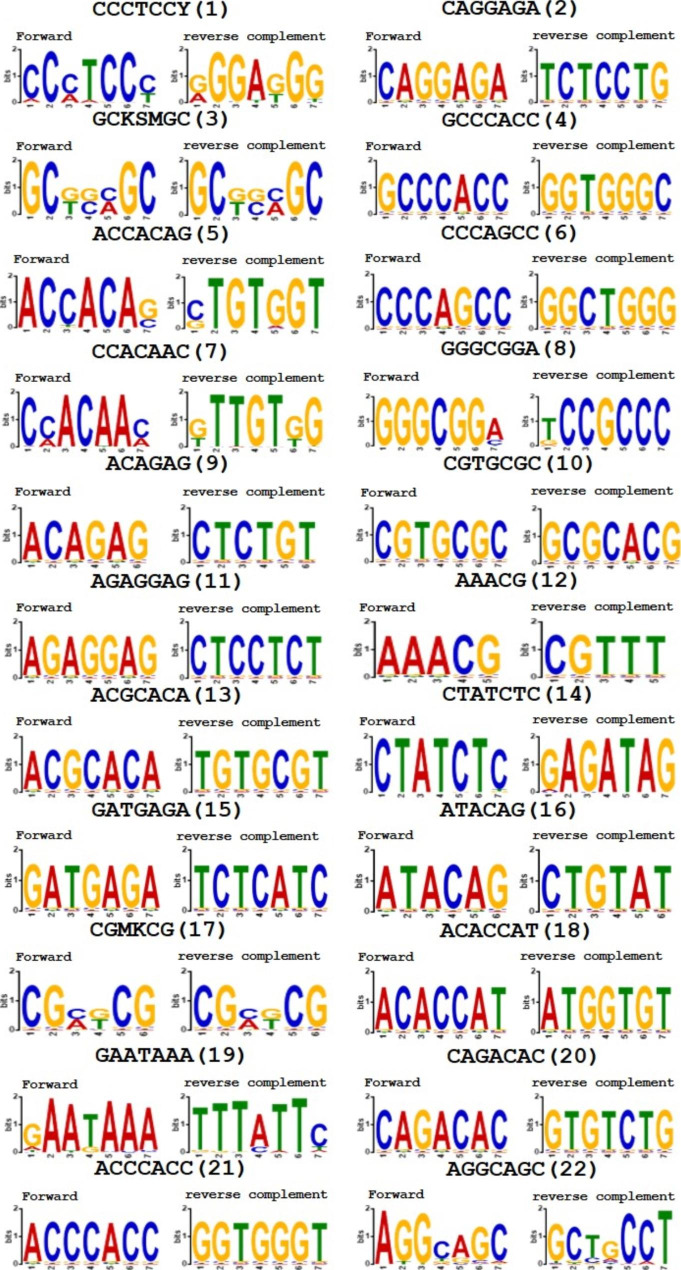
Analysis of the motifs potentially binding by BpERF2 using the MEME method. The unique clean reads were aligned using MEME program to identify the conserved motifs. In total, 22 motifs were identified

To further study whether BpERF2 can bind other motifs, the insertion sequences were assembled to obtain the unique insertion sequences, which were aligned using the MEME program, and 22 motifs were obtained (Fig. 2), representing the motifs that are potentially bound by BpERF2.

### Yeast one hybrid (Y1H) analysis of the motifs bound by BpERF2

**Fig. 3:**
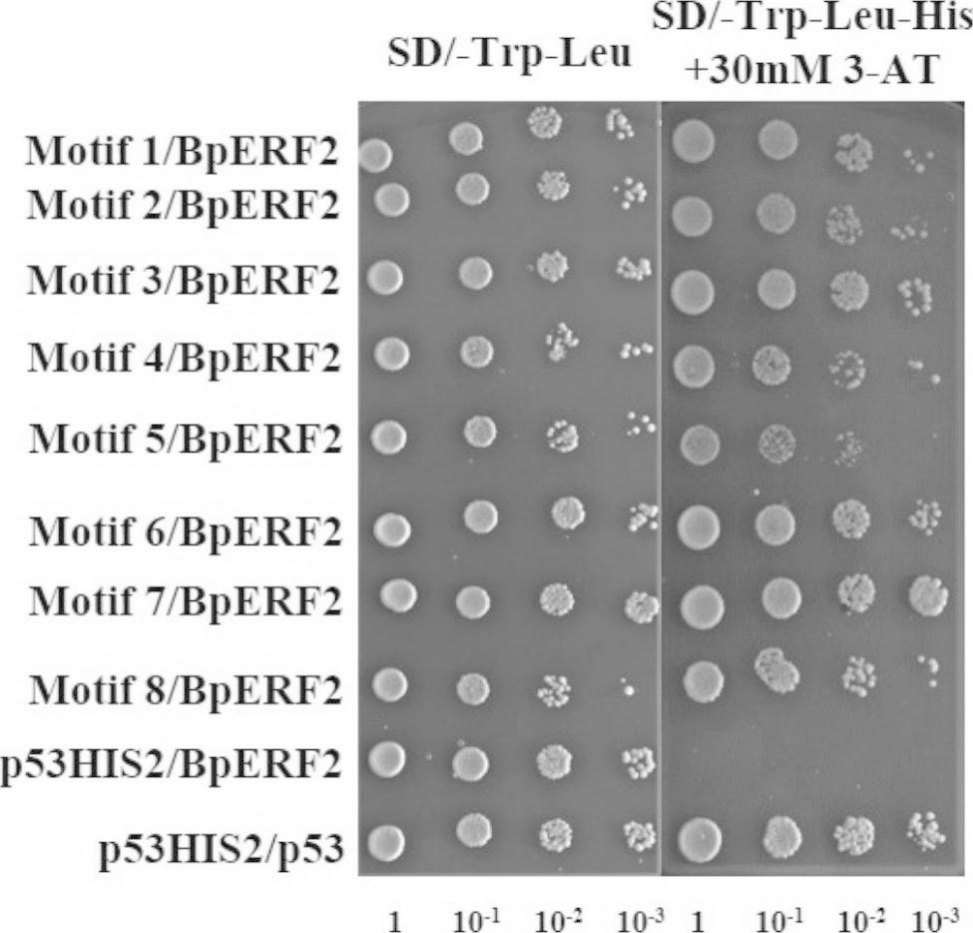
Analysis of the bindings of BpERF2 to the selected motifs using the yeast one hybrid system. Eight motifs were randomly selected, three tandem copies of each motif were cloned into pHIS2 and analyzed by using the yeast one hybrid system

Y1H was first used to study whether the identified motifs (Fig. 3) could bind by BpERF2. Eight motifs were randomly selected from the 22 motifs for analysis. Three tandem copies of motifs were separately cloned into pHIS2 vector and used for Y1H analysis. The identified motifs were then interacted with BpERF2. The Y1H results showed that BpERF2 could bind to all the eight selected motifs.

### EMSA analysis

**Fig. 4:**
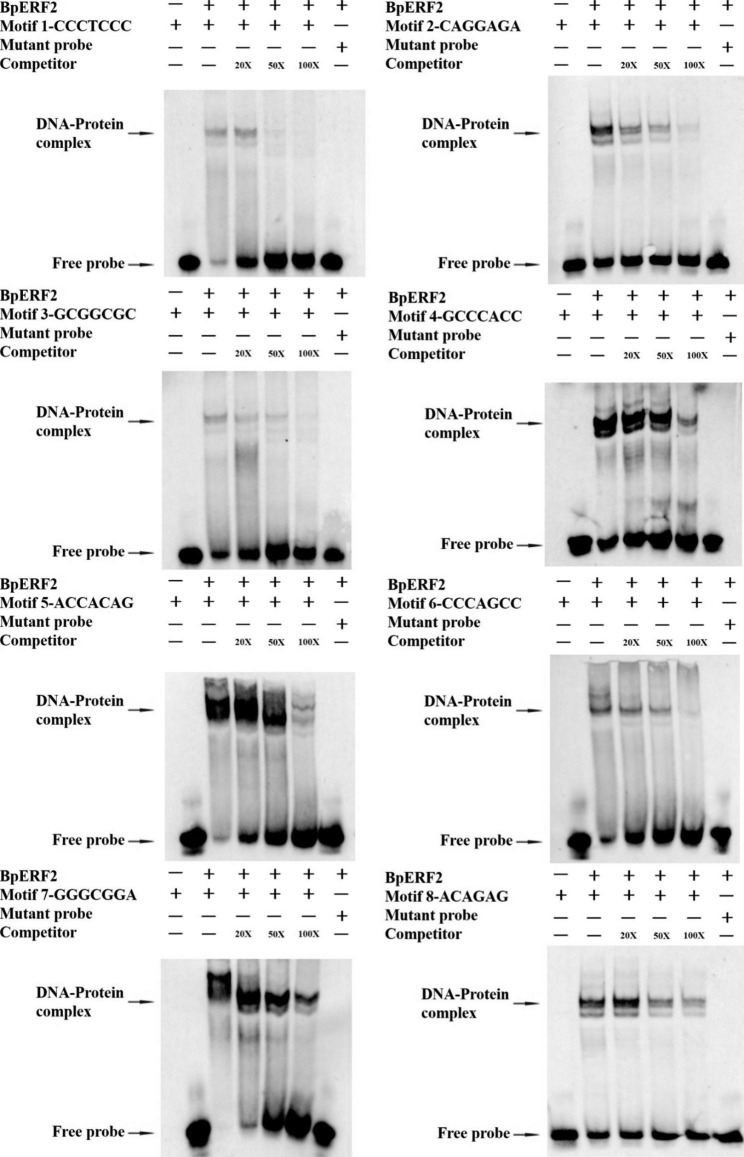
EMSA verification of the bindings of the selected motifs with BpERF2. The eight selected study motifs were used for EMSA analysis. The competitor for the labeled probe was tested by adding 20, 50, and 100-fold excess of the same unlabeled probe. The biotin-labeled, mutated probes were used as the negative control. Arrows indicate the free probes and the DNA-protein complexes

To further validate the binding of BpERF2 to these identified motifs, EMSA was performed. The EMSA results indicated that the BpERF2 protein could interact with all eight selected motifs, to displaying retarded binding bands (Fig. 4). In addition, the binding signals were gradually decreased when the competitor (unlabeled probe) was continually added to the system. However, no retarded binding bands were detected when using the mutant probe (Fig. 4). Therefore, these results showed that all the studied motifs could be bound by BpERF2, which was consistent with the results of Y1H. The Y1H and EMSA results together suggested that these eight motifs could be bound by BpERF2.

### Chromatin immunoprecipitation (ChIP) analysis

**Fig. 5:**
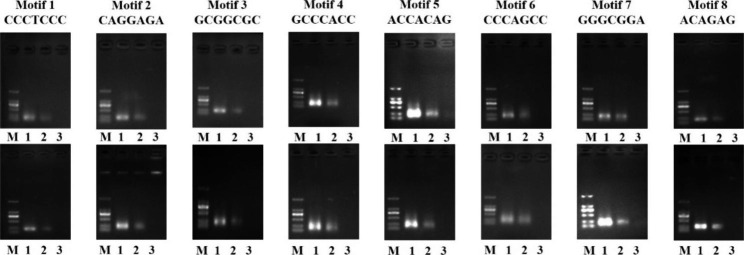
ChIP analysis to verify the bindings of BpERF2 to the selected motifs. The eight selected study motifs were further screened in the promoters of genes from birch, and the truncated promoters containing only the studied motif and without any other identified 21 motifs in this study or GCC-Box and DRE/CRT motifs were selected for ChIP analysis. M: DL2000 marker; 1: Input; 2: the PCR product using ChIP product as PCR template (ChIP+); 3: the PCR product with the template using no antibody for immunoprecipitation (ChIP-)

To further confirm whether these identified motifs and the bindings of BpERF2 to these motifs are really occurred in birch cells. In total, 8 motifs studied in MESA were further studied. For each of these motifs, two promoters (containing only this motif without any other identified motifs and GCC-Box and DRE/CRT) were selected for ChIP analysis. The ChIP-PCR were analyzed using agarose electrophoresis. The results showed that all the ChIP-PCR product (ChIP+) can be amplified with the aim bands (Fig. 5); meanwhile, the negative control (using no antibody for immunoprecipitation) failed in amplifying the aim bands, and Input (positive control) can also amplify the aim bands (Fig. 5). These results suggested that the binding of BpERF2 to these motifs were really occurred in birch cells.

## Discussion

Previously, we built a TF-Centered Y1H method, which could identify the motifs bound by a TF of interest [7]. In the present study, we improved this TF-Centered Y1H method on library construction, positive clones harvested (all positive clones were harvested for analysis), DNA sequencing and DNA motif analysis (Fig. 1). Compared with the previous method, the following aspects were improved: (1) The prey library used for TFCentered Y1H had been improved to incorporate many randomized motif sequences; (2) high-throughput sequencing was used to determine the insertion sequences; (3) bioinformatic analysis was performed to determine the conserved motifs, and the DNA motifs were identified with high throughput, enabling that the motifs bound by a TF can be identified comprehensively.

### The prey library had been improved

In the previous study, the prey library was constructed by inserting a DNA sequence into vector pHIS2 using T4 DNA ligase, which has low efficiency compared with recombination-mediated cloning in yeast. Moreover, in previous study, the insertion sequence is a truncated sequence of pHIS2 containing a random DNA sequence between *Sma* I sites; therefore, there were some repeat sequences in the constructed prey library (Supplementary Fig. 1). However, the newly constructed library comprises an array of short random DNA sequences inserted into *Sma* I site (“GGG” and “CCC”), and has no repeat sequences (Supplementary Fig. 1). The new prey library contains about 150,000 colonies and is a saturated library. However, the previous library contained only about 30,000 clones. Therefore, compared with the previous library, this library had the following advantages: (1) No repeat sequences in the prey library, making the library easy to analyze; (2) it harbors nearly all types of insertion sequences; therefore, it could detect more motifs than the previous library.

The reason why we constructed the 7 bp random insertion library is that this library contains only 16,384 independent sequences, and can be easily constructed with a saturated library. In addition, many known motifs are less than 8 bp, and therefore a 7 bp sequence can meet the requirements of most studies. The limitation of 7 bp random insertion is that it cannot detect the cis-acting element more than 7 bp. However, of course, if needed, a longer insertion library can be constructed using the method described in this article.

### TF-centered Y1H combined with high-throughput sequencing is powerful to determine TF binding motifs

The previous TF-Centered Y1H found it difficult to identify the motifs bound by a TF comprehensively. In the previous study, the positive clones needed to be picked onebyone and sequenced using Sanger sequencing. At the same time, many motifs may have different bases at one position of a motif; however, this kind of sequence is difficult to be discovered using Sanger sequencing one-by-one. In the present study, combined with high-throughput sequencing, the newly developed method could identify the motifs more efficiently, and could be used to discover the motifs with different bases at one position of a motif (Fig. 2). Therefore, this method enables us to comprehensively understand the motifs bound by as TF of interest. Another advantage of usage of high-throughput sequencing method is that can eliminate empty vector contamination easily, for the reason that only insertion sequences can be retrieved and analyzed. Therefore, this method can even adapt the prey library with low quality.

Therefore, the TF-Centered Y1H combined with high-throughput sequencing technology provides a tremendous increase in the throughput of DNA-Protein interaction identification compared with the previous TF-Centered Y1H method. In theory, it can detect all the DNA-Protein interactions (if the insertion sequence in the prey library is large enough) and could be used for all the proteins from any organism species.

All the sequences obtained using TF-Centered Y1H can be bound by the target TF in theory. However, there may inevitably be some false positives in TF-Centered Y1H. Commonly, these false positive clones will not be too many. Therefore, the sequence with more repetitions is more likely to be the cis-acting elements bound by the target TF. In this study, to found the sequence with more repetitions, MEME was used, and the sequences that potentially bound by BpERF2 were identified (Fig. 2). ChIP, Y1H and EMSA assays all showed that the identified sequences could be bound by BpERF2 in vivo or in vitro (Figs. 3, 4 and 5), suggesting that MEME analysis was reliable to determine the motifs. In this study, 30 mM 3-AT was used for selection. For the reason that selection of library needs moderate selection strength to obtain ideal results. Generally, 30 mM 3-AT is a moderate selection strength, which can be used to screen the library efficiently [7].

This improved TF-Centered Y1H can efficiently identify the motifs bound a target TF; however, it still has some limitation. One is that it can not detect the cis-acting element with discontinuous. The other limitation is that as many reads were generated, it needs a powerful tool to identify the motifs. Although MEME analysis can work well to identify the conserved motifs, it cannot be guarantee that all the sequences bound by the target TF could be detected using MEME analysis. For instance, the GCC-Box and DRE/CRT motifs that had been included in the reads with a relative high frequency, but they cannot be detected by MEME analysis according to the E-value. Therefore, some parameters need to be adjusted in MEME analysis or a suitable tool to identify the conserved motifs needs to be exploited for TF-Centered Y1H.

### The application of TF-centered Y1H

This method can be used to detect the motifs bound by a defined TF systematically, which will help to reveal the function of TFs and the mechanisms of DNA-protein interactions. Although the all the possible protein and DNA interactions can be quickly determined using this method, it cannot indicate whether the DNA-protein interaction really occurs in the organism and the identified motif has a biological function or not. Therefore, for the biological significance, the identified motifs need to be screened in the promoters of genes to determine their overall occurrence. ChIP-PCR, based on transient transformation [8], can be performed to study whether the target TF can bind with these motifs. Moreover, this method could be combined with ChIP-seq to investigate the motifs. First, the motifs potentially bound by the TF of interest could be identified comprehensively using TF-Centered Y1H, and whether these motifs whether can map with the peaks identified from ChIP-seq or not (i.e., those peaks containing the motifs or not) could be analyzed, which will illustrate the role of the motifs identified by TF-centered analysis in gene expression regulation, and allow cross-confirmation between ChIP-Seq and TF-Centered Y1H.

### The motifs bound by BpERF2

The reason that we used BpERF2 in this study is that we found it can be induced by drought stress; at the same time, BpERF2 can regulate a serial of genes to confer drought tolerance [9]. Therefore, it is necessary to investigate the motifs bound by BpERF2, which will be helpful to reveal the drought tolerance mediated by BpERF2 in depth. Previous studies showed that ERF protein can bind to the different motifs, including GCC-Box and DRE/CRT motifs [10, 11]. In the present study, we found that BpERF2 also can bind 22 other motifs in addition to binding to GCC-Box and DRE/CRT motifs. These results indicated that the ERF transcription factor might have more binding motifs than previously assumed, and revealing these motifs is helpful to investigate the function of TFs. Therefore, to reveal the function of TFs in depth, it is necessary to study their binding motifs using this newly developed method.

## Conclusion

In the present study, we improved the TF-Centered yeast one hybrid technology by using the yeast recombination method to construct a prey library and combine it with high-throughput sequencing to completely determine the motif. These improvements substantially increased the efficiency to detect motifs bound by a defined TF, because this method can not only detect the motifs with high-throughput sequencing, but also can determine different types of motifs. Therefore, this method makes it is possible to reveal the motifs bound by a TF of interest comprehensively.

## Materials and methods

### Construction of a random insertion prey library using recombination in yeast

To construct an DNA random insertion library, a single strand DNA sequence (125 nt in length) (single strand insertion sequence, SsInS) with a 7 nt random sequence that was inserted at the *Sma* I digestion site to form “CCCNNNNNNNGGG” in the vector of pHIS2 (Clontech, CA, USA) was designed (Table 1). At the same time, a primer (complementary primer of SsInS, termed as CP primer) (Table 1) was designed. Both SsInS and CP primer were synthesized chemically by Shanghai Sangon Biotechnology (China). The complementary DNA strand of SsInS was synthesized using SsInS as template and CP as the initial primer to form DsInS (Double strand insertion sequence, DsInS). In detail, DNA synthesis reaction was carried out using 5 µl of SsInS (10 µM), 6 µl of CP10 µM), 2 µl 10 × PCR buffer, 0.5 µl Taq with sterile distilled water to a total volume of 20 µl. The reaction thermal profile for synthesizing DsInS was: 94 °C for 3 min and then at 68 °C for 3 min.

pHIS2 linearized by digestion of *Sma* I, and 3 µl of linearized pHIS2 (0.5 µg/µl), together with 2.0 µg of DsInS, 10 µl of Herring Testes Carrier DNA (denatured), add 600 µl of competent Y187 yeast cells to the DNA, gently mix by vortexing and add 1.5 ml PEG/LiAc Solution. The procedure of yeast transformation was according to the user manual of Yeastmaker™ Yeast Transformation System 2 (Clontech) with large scale transformation. The transformed cells were grown on SD/-His/-Leu/-Trp (TDO) medium supplied with 30 mM 3-AT (3-Amino-1, 2, 4-triazole) at 30 °C. After the colonies grew, the plates were washed with 10 ml of potato dextrose agar (PDA) liquid buffer, and all the wash buffer were collected together to form the random DNA insertion library, which was used as the prey library in TF-Centered Y1H.

### Construction of the bait vector

To construct the bait vector for TF-Centered Y1H, the coding sequence of *BpERF2* (birch ethylene-responsive factor 2) was amplified and cloned into vector pGADT7-Rec2-ERF2 using the yeast recombination method. The PCR reaction to amplify *BpERF2* comprised 0.5 µl of cDNA template, 0.5 µM of primers BpERF2-F and BpERF2-R (Table 1), 2.5 mM each dNTP, 2 µl of 10 × buffer, 0.5 U Taq, with sterile distilled water to a volume of 20 µl. The PCR thermal profile was 94 °C for 3 min; followed by 25 cycles of 94 °C for 30 s, 58 °C for 30 s, and 72 °C for 2 min. The PCR product was purified using a Column PCR product purification kit (Qiagen, Hilden, Germany) according to the supplier’s protocol. pGADT7-Rec2 was linearized using *Sma* I. The linearized pGADT7-Rec2 and the PCR product of *BpERF2* were cotransformed into Y187 using the Yeastmaker™ Yeast Transformation System 2 with small scale transformation to generate the recombined pGADT7-Rec2-ERF2. The recombined pGADT7-Rec2-ERF2 was isolated from yeast cells using an Easy Yeast Plasmid Isolation Kit (PT4073–1, Clontech), and was transformed into DH5α *Escherichia coli*. The recombined pGADT7-Rec2-ERF2 vector was isolated from *E. Coli* usin*g* E.Z.N.A.TM Plasmid Mini Kit (OMEGA).

### Screening the random DNA insertion library

The recombined vector pGADT7-Rec2-ERF2 was transformed into the yeast strain harboring the random DNA insertion prey library using Yeastmaker™ Yeast Transformation System 2. The transformed cells were selected on SD/–His/–Leu/–Trp + 30 mM 3-aminotriazole (3-AT). The yeast transformants on each plate were harvested by washing using YPD Plus liquid medium, and pooled together to form the hybridization library. The plasmid was isolated using an Easy Yeast Plasmid Isolation Kit (Takara, Dalian, China), and was used as template for PCR amplification using primers P1 and P2 (Table 1). The PCR reaction system comprised 1 µl of plasmid (10 ng), 0.5 µM each of P1 and P2 primers, 2.5 mM of each dNTP, 2 µl of 10 × buffer, 0.5 U Taq, with sterile distilled water to a volume of 20 µl. The PCR thermal profile was 94 °C for 3 min; followed by 30 cycles of 94 °C for 30 s, 58 °C for 30 s, and 72 °C for 2 min.

### High-throughput sequencing

The PCR products were sequenced on the Illumina Hiseq 2000 (Illumina, San Diego, CA, USA) with 150 bp paired-end reads by Novogene Biotech (Tianjin, China). The raw data were filtered using the Trimmomatic v0.30 program and FastQC to remove low-quality reads, including the adaptor reads, reads with N > 5% (N indicates a base that cannot be determined), and sequences with Q ≤ 10, to obtain the clean reads. After obtaining the clean reads, the random insertion sequences were extracted from these reads. The insertion reads were assembled to obtain the unique insertion reads, and then analyzed using MEME program (https://meme-suite.org/meme/tools/streme) with default parameters to identify the potential sequences bound by BpERF2.

### Verification of the binding of motifs to TF using yeast one hybrid

Three tandem copies of the different cis-acting element were respectively cloned into pHIS2 vector as reporter vectors, and the primers used were shown as Supplementary Table 1. The reporter vector was transformed into yeast strain Y187 together with pGADT7-Rec2-ERF2, and were plated on DDO(SD/-Leu/-Trp) and TDO (SD/-Leu/-Trp/-His) media supplemented with 30 mM 3-AT for yeast one hybrid.

### Electrophoretic mobility shift assay (EMSA)

The full coding sequence (CDS) of *BpERF2* was amplified with specific primers (Supplementary Table 2), and was cloned into the pMAL-5 vectors (NEB, Ipswich, MA, USA) to fuse with the maltose-binding protein (MBP) to generate the vector MBP-ERF2. Vector MBP-ERF2 was transformed into *E. coli* ER2523 and was purified according to the instruction manual of the pMAL™ Protein Fusion & Purification System (NEB). The probes were synthesized and labeled with biotin (Supplementary Table 3). Different concentrations of non-labeled probe were added to the reactions for competition. The EMSA was carried out using a Chemiluminescent EMSA kit (Beyotime, Jiangsu, China). The sequences of the probes are shown as Supplementary Table 3.

In brief, the *E. coli* cells were harvested and resuspended in a buffer containing 20 mM Tris-HCl, 0.2 M NaCl, 1 mM EDTA (pH 8.0), and then sonicated to release the protein. Amylose resin (NEB) was used to isolate the protein by affinity and the fusion protein was eluted using maltose. SDSPAGE was used to check the recombinant BpERF2 protein. The probes were labeled using an EMSA Probe Biotin Labeling Kit (Beyotime, China) following the user manual. The same unlabeled probe served as the competitor. The mutated probes were also biotin labeled and served as negative controls. The probes were incubated with the recombinant BpERF2 protein and analyzed using the Chemiluminescent EMSA kit (Beyotime).

### ChIP-PCR analysis

To determine whether the identified interaction was actually occurred in plants, ChIP-PCR was performed. *BpERF2* was fused with Flag tag in frame, and transient transformed into birch for overexpression. The transformation procedure was followed by Zang et al. [12]. After transformed for 48 h, the plants were harvested and was used for ChIP analysis. ChIP was conducted with anti-Flag antibody according to the method of Zhao et al. [13], and the ChIP product was used as PCR template for ChIP-PCR. The identified motifs were screened in the promoters of genes from the birch genome, and the truncated promoters containing only one of the identified motifs, and without any known motif bound by ERF (such as GCC-Box and DRE/CRT) were used for ChIP-PCR. The PCR reaction system comprised 1 µl of ChIP product, 0.5 µM each of primers, 2.5 mM of each dNTP, 2 µl of 10 × buffer, 0.5 U Taq, with sterile distilled water to a volume of 20 µl. The PCR thermal profile was 94 °C for 3 min; followed by 30 cycles of 94 °C for 30 s, 58 °C for 30 s, and 72 °C for 2 min.

## Electronic supplementary material

Below is the link to the electronic supplementary material.

Supplementary Material 1: Supplementary Fig. 1 Comparison of pHIS2, the newly constructed pHIS2 prey library, and the previously constructed pHIS2 prey library. The green line indicates the two flanking sequences of the *Sma* I sites. (1) The map of pHIS2. (2) The prey library built in this study. (3) The prey library built in the previous study, which contains two types of pHIS2. The insertion is underlined.

Supplementary Material 2: Supplementary Table 1. The sequences used for yeast one hybrid.

Supplementary Material 3: Supplementary Table 2. The primers for the construction of the recombination Vector MBP-ERF2.

Supplementary Material 4: Supplementary Table 3. The sequences of the probes used in EMSA assay.

Supplementary Material 5: Supplementary Table 4. The primers used in ChIP analysis.

Supplementary Material 6

## Data Availability

All data generated or analyzed during this study had been included in this published article and its supplementary information files. The GenBank accession number of BpERF2 was MK112037. The RNA-Seq data in this manuscript also had been deposited in a public database, which can be accessed at the website https://figshare.com/articles/dataset/Short_DNA_sequence_recognized_by_ERF2/19164662 with the DOI number of 10.6084/m9.figshare.19164662.v1.

## References

[1] Ferraz RAC, Lopes ALG, da Silva JAF, Moreira DFV, Ferreira MJN, de Coimbra A (2021). DNA-protein interaction studies: a historical and comparative analysis. Plant Methods.

[2] Lin JJ, Grosskurth SE, Harlan SM, Gustafson-Wagner EA, Wang Q (2007). Characterization of cis-regulatory elements and transcription factor binding: gel mobility shift assay. Methods Mol Biol.

[3] Reece-Hoyes JS, Diallo A, Lajoie B, Kent A, Shrestha S, Kadreppa S (2011). Enhanced yeast one-hybrid assays for high-throughput gene-centered regulatory network mapping. Nat Methods.

[4] Sánchez-Romero, Olivenza DR, Gabriel G, Josep C, MA (2020). Contribution of DNA adenine methylation to gene expression heterogeneity in Salmonella enterica. Nucleic Acids Res.

[5] Oliveira R, Pinho E, Sousa AL, Destefano JJ, Azevedo NF, Almeida C (2021). Improving aptamer performance with nucleic acid mimics: de novo and post-SELEX approaches. Trends Biotechnol.

[6] John S, Reece-Hoyes AJ, Marian (2012). Yeast one-hybrid assays: a historical and technical perspective. Methods.

[7] Ji X, Wang L, Nie X, Lin H, Wang Y (2014). A novel method to identify the DNA motifs recognized by a defined transcription factor. Plant Mol Biol.

[8] Zang D, Wang L, Zhang Y, Zhao H, Wang Y (2017). ThDof1.4 and ThZFP1 constitute a transcriptional regulatory cascade involved in salt or osmotic stress in Tamarix hispida. Plant Mol Biol.

[9] Wen X, Wang J, Zhang D, Wang YA (2019). Gene Regulatory Network controlled by BpERF2 and BpMYB102 in Birch under Drought Conditions. Int J Mol Sci.

[10] Shinshi H, ‘Masaru Ohme-Takagi (1995). Ethylene-inducible DNA binding proteins that interact with an ethylene-responsive element. Plant Cell.

[11] Zhang Z, Zhang H, Quan R, Wang X, Huang R (2009). Transcriptional regulation of the ethylene response factor LeERF2 in the expression of ethylene biosynthesis genes controls ethylene production in tomato and tobacco. Plant Physiol.

[12] Zang D, Wang L, Zhang Y, Zhao H, Wang Y. ThDof1.4 and ThZFP1 constitute a transcriptional regulatory cascade involved in salt or osmotic stress in Tamarix hispida. Plant Mol Biol. 2017;94(4–5):495–507. 10.1007/s11103-017-0620-x 28578496

[13] Zhao H, Li H, Jia Y, Wen X, Guo H, Xu H, Wang Y (2020). Building a robust chromatin immunoprecipitation method with substantially improved efficiency. Plant Physiol.

