# Supplementary figures and images for: Building an improved transcription factor-centered yeast one hybrid system to identify DNA motifs bound by protein comprehensively
Source: BMC Plant Biol. 2023 May 4;23:236. doi: 10.1186/s12870-023-04241-8 (PMC10158250; doi:10.1186/s12870-023-04241-8)

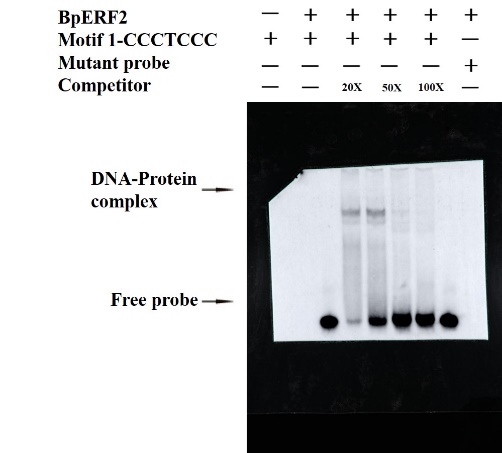

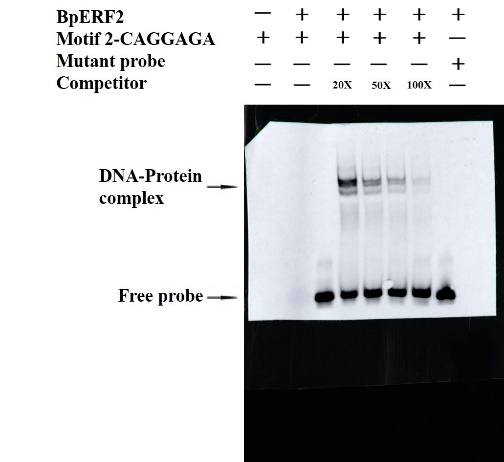


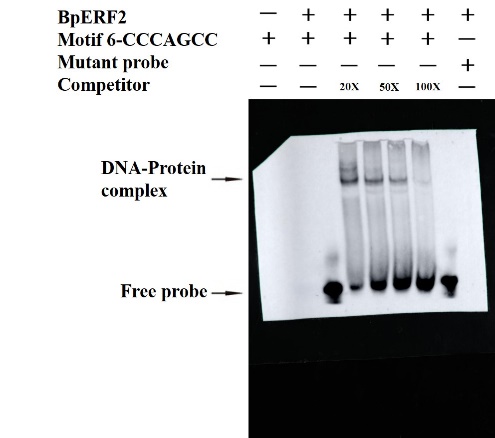

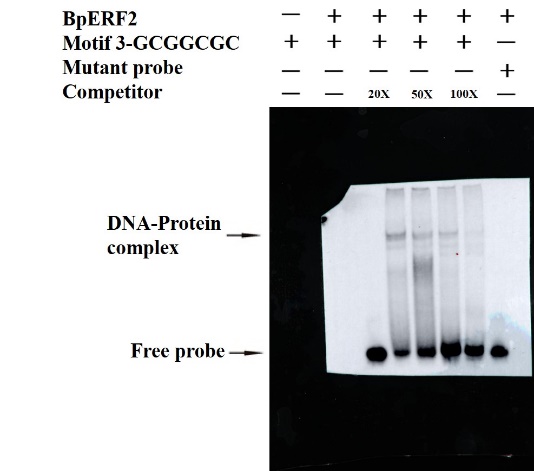

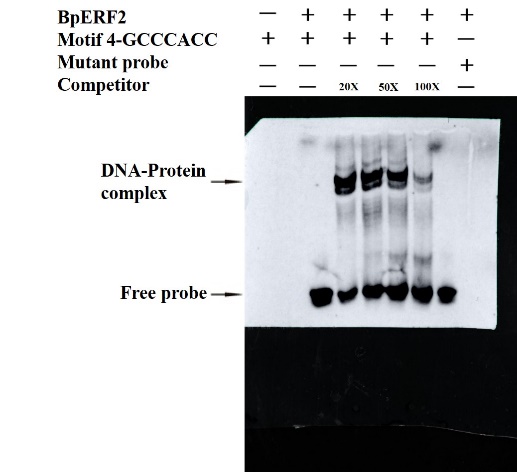


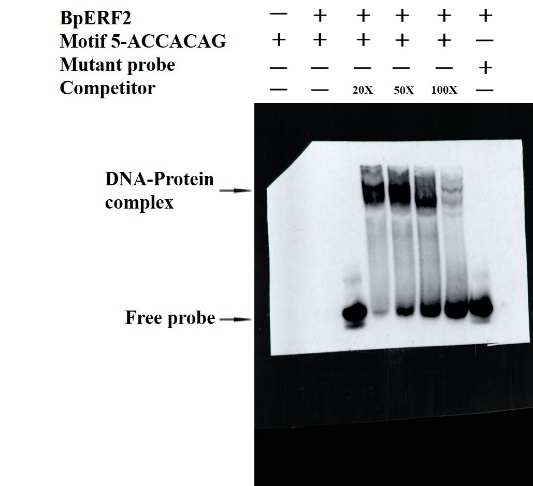

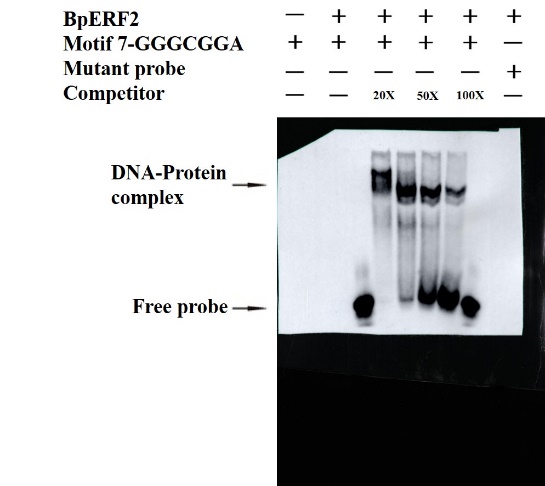

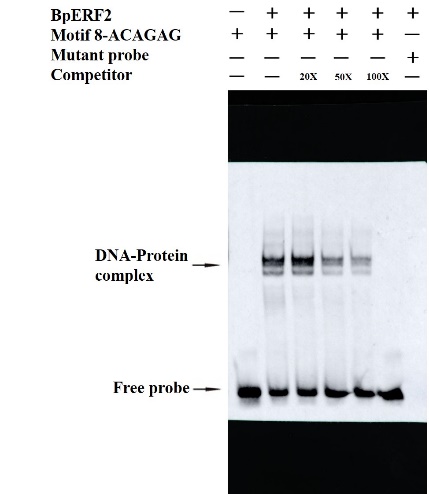

Supplement: Supplementary file 1 — Supplementary Material 1: Supplementary Fig. 1 Comparison of pHIS2, the newly constructed pHIS2 prey library, and the previously constructed pHIS2 prey library. The green line indicates the two flanking sequences of the Sma I sites. (1) The map of pHIS2. (2) The prey library built in this study. (3) The prey library built in the previous study, which contains two types of pHIS2. The insertion is underlined. [file 12870_2023_4241_MOESM1_ESM.docx]
