# Supplementary material for: Building an improved transcription factor-centered yeast one hybrid system to identify DNA motifs bound by protein comprehensively
Source: BMC Plant Biol. 2023 May 4;23:236. doi: 10.1186/s12870-023-04241-8 (PMC10158250; doi:10.1186/s12870-023-04241-8)
Supplement: Supplementary file 2 — Supplementary Material 2: Supplementary Table 1. The sequences used for yeast one hybrid. [file 12870_2023_4241_MOESM2_ESM.docx]

CCCGGG

**(1) pHIS2 vector**

**(2) The constructed pHIS2 prey library in this study**

Insertion

CCCNNNNNNNGGG

**(3) The previous pHIS2 prey library**

Insertion

CANNNNNNC

CCC

GGG

Insertion

CC(T)NNNNNN

CCC

GGG

**Supplementary Fig. 1 Comparison of pHIS2, the newly constructed pHIS2 prey library, and the previously constructed pHIS2 prey library.** The green line indicates the two flanking sequences of the *Sma* I sites. (1) The map of pHIS2. (2) The prey library built in this study. (3) The prey library built in the previous study, which contains two types of pHIS2. The insertion is underlined.
