# Supplementary material for: Building an improved transcription factor-centered yeast one hybrid system to identify DNA motifs bound by protein comprehensively
Source: BMC Plant Biol. 2023 May 4;23:236. doi: 10.1186/s12870-023-04241-8 (PMC10158250; doi:10.1186/s12870-023-04241-8)
Supplement: Supplementary file 3 — Supplementary Material 3: Supplementary Table 2. The primers for the construction of the recombination Vector MBP-ERF2. [file 12870_2023_4241_MOESM3_ESM.docx]

**Supplementary Table 1** **The sequences used for yeast one hybrid**

| Names | Sequence (5′-3′) |
| --- | --- |
| Motif1-F | AATTCCCCTCCCCCCTCCCCCCTCCCCCGC |
| Motif1-R | GGGGGAGGGGGGAGGGGGGAGGGG |
| Motif2-F | AATTCCAGGAGACAGGAGACAGGAGACCGC |
| Motif2-R | GGTCTCCTGTCTCCTGTCTCCTGG |
| Motif3-F | AATTCGCGGCGCGCGGCGCGCGGCGCCCGC |
| Motif3-R | GGGCGCCGCGCGCCGCGCGCCGCG |
| Motif4-F | AATTCGCCCACCGCCCACCGCCCACCCCGC |
| Motif4-R | GGGGTGGGCGGTGGGCGGTGGGCG |
| Motif5-F | AATTCACCACAGACCACAGACCACAGCCGC |
| Motif5-R | GGCTGTGGTCTGTGGTCTGTGGTG |
| Motif6-F | AATTCCCCAGCCCCCAGCCCCCAGCCCCGC |
| Motif6-R | GGGGCTGGGGGCTGGGGGCTGGGG |
| Motif7-F | AATTCGGGCGGAGGGCGGAGGGCGGACCGC |
| Motif7-R | GGTCCGCCCTCCGCCCTCCGCCCG |
| Motif8-F | AATTCACAGAGACAGAGACAGAGCCGC |
| Motif8-R | GGCTCTGTCTCTGTCTCTGTG |
