# Supplementary material for: Building an improved transcription factor-centered yeast one hybrid system to identify DNA motifs bound by protein comprehensively
Source: BMC Plant Biol. 2023 May 4;23:236. doi: 10.1186/s12870-023-04241-8 (PMC10158250; doi:10.1186/s12870-023-04241-8)
Supplement: Supplementary file 4 — Supplementary Material 4: Supplementary Table 3. The sequences of the probes used in EMSA assay. [file 12870_2023_4241_MOESM4_ESM.docx]

**Supplementary Table 2 The primers for the construction of the recombination Vector MBP-ERF2**

| Names | Sequence (5′-3′) |
| --- | --- |
| MBP**-**ERF2-F | CGGGATCCATGTGTGGGGGTGCTATCAT |
| MBP-ERF2-R | GGAATTCCTAATACATGAGCTTCAGTT |
