# Supplementary material for: Building an improved transcription factor-centered yeast one hybrid system to identify DNA motifs bound by protein comprehensively
Source: BMC Plant Biol. 2023 May 4;23:236. doi: 10.1186/s12870-023-04241-8 (PMC10158250; doi:10.1186/s12870-023-04241-8)
Supplement: Supplementary file 5 — Supplementary Material 5: Supplementary Table 4. The primers used in ChIP analysis. [file 12870_2023_4241_MOESM5_ESM.docx]

**Supplementary Table 3 The sequences of the probes used in EMSA assay**

| Names | Sequence (5′-3′) |
| --- | --- |
| Motif1 probe-F  Motif1 probe-R | GGCGGCCCCTCCCTTCCCTCCCTTCCCTCCCG GCGGCGGGAGGGAAGGGAGGGAAGGGAGGGG |
| Motif2 probe-F  Motif2 probe-R  Motif3 probe-F  Motif3 probe-R  Motif4 probe-F  Motif4 probe-R  Motif5 probe-F  Motif5 probe-R  Motif6 probe-F  Motif6 probe-R  Motif7 probe-F  Motif7 probe-R  Motif8 probe-F  Motif8 probe-R  mutant probe-F  mutant probe-R | GGCGGCCAGGAGATTCAGGAGATTCAGGAGAG  GCGGCTCTCCTGAATCTCCTGAATCTCCTGG  GGCGGCGCGGCGCTTGCGGCGCTTGCGGCGCG  GCGGCGCGCCGCAAGCGCCGCAAGCGCCGCG  GGCGGCGCCCACCTTGCCCACCTTGCCCACCG  GCGGCGGTGGGCAAGGTGGGCAAGGTGGGCG  GGCGGCACCACAGTTACCACAGTTACCACAGG  GCGGCCTGTGGTAACTGTGGTAACTGTGGTG  GGCGGCCCCAGCCTTCCCAGCCTTCCCAGCCG  GCGGCGGCTGGGAAGGCTGGGAAGGCTGGGG  GGCGGCGGGCGGATTGGGCGGATTGGGCGGAG  GCGGCTCCGCCCAATCCGCCCAATCCGCCCG  GGCGGCACAGAGTTACAGAGTTACAGAGG  GCGGCCTCTGTAACTCTGTAACTCTGTG  GGCGGCAAAAAAATTAAAAAAATTAAAAAAAG  GCGGCTTTTTTTAATTTTTTTAATTTTTTTG |
