# Supplementary material for: Building an improved transcription factor-centered yeast one hybrid system to identify DNA motifs bound by protein comprehensively
Source: BMC Plant Biol. 2023 May 4;23:236. doi: 10.1186/s12870-023-04241-8 (PMC10158250; doi:10.1186/s12870-023-04241-8)
Supplement: Supplementary file 6 — Supplementary Material 6 [file 12870_2023_4241_MOESM6_ESM.docx]

**Supplementary Table 4 The primers used in ChIP analysis.**

| Motif sequence | Names | Sequence (5′-3′) |
| --- | --- | --- |
|  | pROKII-ERF2-Flag-F | CTAGTCTAGAATGTGTGGGGGTGCTATCAT |
|  | pROKII-ERF2-Flag-R | CTAGGGATCCCTTGTCATCGTCGTCCTTGTAATCATACATGAGCTTCAGTTGTT |
| Motif1-CCCTCCC | Motif1-c1000-F | AGACATTGATGTCCACTTCT |
|  | Motif1-c1000-R | GATTTGGCTCCCTTGCACGT |
|  | Motif1-c1600-F | GTCGACGCTCATGATGGTTC |
|  | Motif1-c1600-R | CCACTTTAGTGAAGAGAGAG |
| Motif2-CAGGAGA | Motif2-c0050-F | GCGTGGTGAGAGAGAGAGAG |
|  | Motif2-c0050-R | CTCTCTTTCTCACTCCACGC |
|  | Motif2-c0286-F | ACATCTACAAGAAGCGCATG |
|  | Motif2-c0286-R | CGCATTCTCAAATTGTGACT |
| Motif3-GCGGCGC | Motif3-c0582-F | CACGGAACCTCTGGGCCGTG |
|  | Motif3-c0582-R | CTCGACTCCACCGTGCCTCT |
|  | Motif3-c0660-F | TGGCGCGTGGAGCTGATTTC |
|  | Motif3-c0660-R | GCCTCATCGAAGTCTTCACG |
| Motif4-GCCCACC | Motif4-c1098-F | CTCCATAGAAACCACCACCGC |
|  | Motif4-c1098-R | GGAGGGTGGGAAACGGCGTT |
|  | Motif4-c1217-F | CTTCACTCTCGTCTCCTCTC |
|  | Motif4-c1217-R | CAGTGGTGGTCGGAGCAGAC |
| Motif5-ACCACAG | Motif5-c0029-F | GTCCAAGGTGGCCTTTGAGG |
|  | Motif5-c0029-R | GCTCATGTAGTTAGCCTAAAT |
|  | Motif5-c0157-F | AACTTGACCATTAGGCTACC |
|  | Motif5-c0157-R | GGCTGGTTGTTCGATGTGGA |
| Motif6-CCCAGCC | Motif6-c0574-F | CCAACCGTGACCCACGCTGG |
|  | Motif6-c0574-R | GGTCACACCAGACCCCGCCG |
|  | Motif6-c0863-F | ACGTGACCCAAGCTGGTCAC |
|  | Motif6-c0863-R | CAGACCACGGCGGGGTCTGG |
| Motif7-GGGCGGA | Motif7-c0105-F | AATGTTCCGGATGGGTCCTG |
|  | Motif7-c0105-R | GCCTTCTTGCCCTGGACATT |
|  | Motif7-c1162-F | TGTGGATCCAGGCTGACGTG |
|  | Motif7-c1162-R | CCAGCGATCGTACTAGGGAC |
| Motif8-ACAGAG | Motif8-c1074-F | GATGCAGAATTTGGTGGAAC |
|  | Motif8-c1074-R | CCATTCCTTGTAAATGATTC |
|  | Motif8-c1564-F | CTAAGAGCACGTACCCAAAG |
|  | Motif8-c1564-R | CCTTAAAGACTTAAAGTGCA |
